# Remote assessment of exercise capacity in adults with chronic respiratory disease: Safety, reliability and acceptability

**DOI:** 10.1177/14799731251318033

**Published:** 2025-01-29

**Authors:** Narelle S Cox, Simone Dal Corso, Angela T Burge, Janet Bondarenko, Jaycie Perryman, Anne E Holland

**Affiliations:** 1 Respiratory Research@Alfred, School of Translational Medicine, 2541 Monash University, Melbourne Australia; 2 Institute for Breathing and Sleep, Melbourne Australia; 3 Physiotherapy Department, 5392 Alfred Health, Melbourne Australia

**Keywords:** Pulmonary rehabilitation, telehealth, exercise testing, COPD, telerehabilitation

## Abstract

**Objectives:**

To assess the safety, reliability and acceptability of the modified incremental step test (MIST) supervised remotely via videoconferencing in adults with chronic respiratory disease.

**Methods:**

Adults with chronic respiratory disease undertaking pulmonary rehabilitation were invited to undertake the MIST under two testing conditions: in-person supervision and remote supervision via video-conferencing. Test order was randomised.

**Results:**

38 participants (*n* = 18 Female; mean (SD) age 68 (10) years; 56% chronic obstructive pulmonary disease) undertook two MIST evaluations. There was excellent agreement between tests for total step count (ICC_2,1_ 0.93, 95%CI 0.86 to 0.96), despite higher counts with in-person supervision (MD 12 steps, 95%CI 1 to 24). There was very good agreement, and no difference between tests, for nadir oxygen saturation (ICC_2,1_ 0.797, 95%CI 0.643 to 0.889) and peak heart rate (ICC_2,1_ 0.782, 95%CI 0.620 to 0.880). Participant satisfaction with telehealth was high, and confidence was not different between testing conditions. There were no adverse events and remote testing was acceptable to participants.

**Discussion:**

In this single centre cohort study MIST supervised remotely via video-conferencing was safe, reliable and acceptable to people with chronic respiratory disease.

## Introduction

Pulmonary rehabilitation is a proven treatment for people with chronic respiratory disease that improves outcomes of importance to patients, in particular physical function and symptoms, and leads to fewer hospital admissions.^1^ A program of exercise and education, pulmonary rehabilitation is typically conducted in-person, as an outpatient, at a healthcare centre.^1^ Delivered in groups over 8-12 weeks, participants are required to attend the centre at least twice weekly.^2^ Yet, due to patient and health system barriers to accessing programs, particularly relating to travel, transport and disability,^3,4^ pulmonary rehabilitation is grossly underutilised.^5^ In recent years, COVID-19 related restrictions further limited access to outpatient pulmonary rehabilitation.

Home-based models of pulmonary rehabilitation, delivered over the phone or Internet (i.e. telerehabilitation), have demonstrated equivalent outcomes to centre-based programs, with higher completion rates.^6,7^ However, international recommendations^8^ stipulate a centre-based assessment of exercise capacity prior to pulmonary rehabilitation for individualised, accurate and safe prescription of exercise training. Thus, barriers to centre attendance for assessment may further limit access to pulmonary rehabilitation. A number of functional tests, including the 4-m gait speed test and 1-min sit-to-stand test have been proposed as surrogate markers for exercise capacity which in a practical sense could be adapted for remote delivery.^9^ However, their agreement with established tests of exercise capacity is variable.^9^ The remote assessment of exercise capacity has largely been limited to exercise tests that preclude the prescription of exercise training, fail to evaluate the true extent of exercise induced oxygen desaturation or do not reliably replicate performance when supervised in-person.^10,11^ A recent study of 28 adults awaiting lung transplantation evaluated remote administration of the six-minute walk test and incremental shuttle walk test, supervised via video-conferencing in the presence of a caregiver.^11^ Despite no adverse events, test performance was consistently better on all in-person supervised testing occasions of a magnitude that approached or exceeded the minimal important difference.^11^ For equitable access to pulmonary rehabilitation to be achieved across all aspects of program delivery, including comprehensive assessment, options for remote testing of exercise capacity that are reliable and allow for accurate and safe exercise prescription are still required.

The modified incremental step test (MIST) is a progressive, externally paced test that is reproducible and produces a maximal exercise response in people with chronic obstructive pulmonary disease (COPD).^12^ We have previously demonstrated the reliability and responsiveness of the MIST undertaken at home by people with COPD, and developed new methods to prescribe exercise training from this test.^13^ However, the presence of a physiotherapist in the home is still required for test administration. Preliminary work in young adults with chronic respiratory diseases suggests it is possible for step tests to be accurately conducted remotely,^14^ and people with COPD have indicated a willingness to use technology to complement pulmonary rehabilitation.^15^ As such, our aim was to assess the safety, reliability and acceptability of the MIST supervised remotely via videoconferencing in adults with chronic respiratory disease.

## Methods

A single-site, prospective cohort study was undertaken. Individuals referred to pulmonary rehabilitation, or who had completed pulmonary rehabilitation within the previous 6 months and not had a hospitalisation for a respiratory exacerbation were invited to participate. To be eligible for inclusion, participants had a primary diagnosis of a chronic respiratory disease including, but not limited to, COPD, Interstitial Lung Disease and bronchiectasis; aged >40 years; and able to read and speak English. Previous experience of using digital technologies was not a requirement for participation. Potential participants were required to have an appropriate adult companion present in the home for the remotely-monitored exercise test, primarily to ensure participant safety and provide assistance in the event of an adverse event. The adult companion was able to provide assistance with technology or equipment set-up if requested by the participant, but was not involved in any aspect of MIST administration. Potential participants were excluded if they had a primary diagnosis of cystic fibrosis, had comorbidities limiting exercise training, required a gait aid for independent mobility on stairs or were deemed to be a falls risk, had cognitive or language difficulties limiting ability to follow instructions and/or were unable to provide written informed consent. The study protocol was approved prospectively by the Alfred Health Human Research Ethics Committee (Project ID 83,705; Reference 47-22).

Participants performed the MIST according to standard protocol under two testing conditions undertaken in random order: in-person supervision and remote supervision. Testing sessions were scheduled approximately 1 week apart.

The MIST protocol has been previously described.^12,13^ The test makes use of a commercially available 20 cm height step, positioned adjacent to a supporting surface such as a handrail. The rate of stepping is externally paced using a digital audio-recording. Beginning at an initial stepping rate of 10 steps/minute, one additional step is added every 30 seconds. Participants continue stepping until volitional exhaustion (breathlessness and/or fatigue).^12^ The supervising clinician may cease the test if the participant is unable to keep pace with the external stepping signal (audio recording) for a period of 15 seconds, or if there is a concern for safety for example unsteady stepping, prolonged oxygen desaturation.^12^ Prior to each test commencement, participants were shown a video demonstrating the stepping procedure (https://homebaserehab.net/assessment/initial-assessment/exercise-capacity/), and completed a short practice (<1 minute) of stepping at the initial cadence (10 steps/minute) for familiarization.

The total number of steps completed in each test was recorded as the primary outcome. A difference of 13 steps or fewer between testing occasions would indicate variability in performance was less than the minimal important difference.^13^ Peripheral oxygen saturation and heart rate were monitored continuously throughout the test (Nonin Palmsat A2500, Nonin Medical, Plymouth, Minnesota, USA), as well as at rest prior to test commencement and during recovery. Participants rated their breathlessness and fatigue according to the modified Borg scale prior to, and at completion of, the test.^16^

In-person supervised MIST evaluation took place in the pulmonary rehabilitation gym at the site of recruitment. A pulmonary rehabilitation clinician, experienced in conducting exercise assessments and exercise training sessions in individuals with chronic respiratory disease, was physically present alongside the patient for testing. Remotely supervised MIST evaluation was undertaken in the patient’s home, with the clinician present via video-conferencing (Zoom, San Jose, California, USA).

All necessary equipment was provided to participants to conduct the remotely-supervised exercise test. This comprised a 4G-enabled tablet computer (Apple iPad, Apple, Cupertino, California, USA), with mobile data, affixed to a stand for video-conferencing; a pulse oximeter (Nonin Palmsat A2500), 20 cm high platform step, and portable speakers. Equipment was delivered to participants home by the research team, however participants were required to set up all equipment for the testing session themselves, with or without the assistance of the support person at their home. Participants were provided with an instruction manual for equipment set-up, and support or troubleshooting was provided remotely by the clinician, if required, via video-conferencing or over the telephone. During exercise testing, the oximeter was worn by participants in a cross-body bag. Monitoring equipment was set up in-line with the tablet camera to be visible on-screen at all times during testing to the clinician located remotely. [Figure S1]

Demographic data including age, height, weight, sex, familiarity with and use of digital devices were established prior to initial testing. Data collected as a part of the standard pulmonary rehabilitation assessment (6-min walk distance; modified Medical Research Council dyspnoea score; Hospital Anxiety and Depression Scale, Chronic Respiratory Disease Questionnaire) were collected from the patient history and used to describe participant characteristics. Familiarity with digital devices was reported using the Internet Use Patterns guide.^17^ Participants were asked to complete the Telehealth Satisfaction Survey (TeSS)^18^ a 10-item survey, answered on a four-point Likert scale, that assesses the use of equipment/technology as well as the patient experience, and has very good internal consistency (Cronbach’s alpha 0.9).^19^ The TeSS is designed to allow adaptation to testing context, enabling evaluation of both in-person and remote testing conditions. It has previously been adapted for use in younger adults with chronic respiratory disease.^20^ In addition, participants were asked to rate their confidence in using the equipment and performing the test using a five-point Likert scale (not at all confident to very confident). After completion of both assessment sessions participants were asked to identify, via a tick-box form, which testing occasion they preferred- in-person; remote video-conferencing, or no preference. The occurrence of adverse events related to test performance was recorded including musculoskeletal injury, trips or falls as a consequence of stepping or presence of equipment in the home, need to call emergency services in response to significant cardiovascular or respiratory event.

Data were analysed using IBM SPSS Statistics V28.0 (IBM Corp., NY, USA). Demographic details, TeSS responses and patient preference data were reported descriptively. Differences in objective outcomes (e.g. number of steps, nadir oxygen saturation, heart rate) between in-person and remote assessments were compared using paired t-tests or Wilcoxon signed rank test as appropriate.

Extent of agreement and bias between ‘in-person’ and ‘remote’ measures of number of steps, pulse oximetry and heart rate were assessed using intra-class correlation coefficients (ICCs) (ICC_2,1_) and Bland–Altman analyses respectively. Data are expressed as mean (standard deviation (SD)) and 95% confidence intervals (95% CI) unless otherwise noted.

The methods of Walter et al^21^ were used to estimate the required sample size based on the reliability (ICC) component. With each participant undertaking two assessments, it was estimated that for 80% power at 0.05 level, a sample size of 35 individuals with stable chronic lung disease was required to reject the null hypothesis that the ICC ≤0.4^22^ An ICC greater than 0.4 would detect at least a moderate relationship between in-person and remote supervision of incremental step test performance.^23^ Up to an additional five people were recruited to account for drop-outs.

## Results

179 people with chronic respiratory disease attending pulmonary rehabilitation were screened for suitability [Figure S2]; 89 potentially eligible candidates were identified, and 45 individuals consented to participate. Seven participants subsequently did not complete any testing sessions due to scheduling conflicts or change in circumstances, leaving a total of 38 participants who completed both in-person and remote MIST testing conditions.

Participants had a mean (SD) age of 68 (10) years and 18 (47%) were female (Table 1). Nearly half (47%) of participants had a diagnosis of COPD and presented with moderate disease severity (forced expiratory volume in one second 71 (22) %predicted (range 24-112 %predicted). At pulmonary rehabilitation enrolment, median [IQR] mMRC score was 1 [1-2] and 6-min walk distance 484 (87) metres. Participants were experienced in the use of technology based on their responses to the Internet Use Patterns guide: all owned a smartphone, laptop or tablet computer (Table 2) and nearly all (92%) accessed the Internet on a daily basis – most commonly for accessing email (95%) and researching topics of interest (92%).

**Table 1.** Participant characteristics.

	*n* = 38
Age	68 (10)
Female, *n* (%)	18 (47%)
Height, m	1.7 (0.1)
BMI, kg.m^2^	26 (4)
Diagnosis, *n* (%)	
COPD	18 (47%)
Bronchiectasis	5 (13%)
ILD	9 (24%)
Asthma	4 (11%)
Other	2 (5%)
Smoking status, *n* (%)	
Current	4 (11%)
Quit	19 (50%)
Never	15 (39%)
Pack year history, median [IQR]	20 [15 to 45]
FEV_1_ %predicted	71 (22)
FVC %predicted	83 (20)
TLCO %predicted	66 (25)
Long term oxygen therapy, *n* (%)	6 (16%)
CRQ	
Dyspnoea domain	16 (7)
Fatigue domain	16 (7)
Emotion domain	32 (11)
Mastery domain	20 (7)
HADS anxiety	
Median [IQR]	4 [2 to 8]
Case, *n* (%)	3 (8%)
HADS depression	
Median [IQR]	5 [2 to 8]
Case, *n* (%)	3 (8%)
mMRC dyspnoea scale	
Median [IQR]	1 [1 to 2]
0/1/2/3/4, *n*	5 / 23 / 4 / 5 / 1
6MWD, m	484 (87)
Days between test 1 and test 2	7 (4)

Data are mean (SD) unless indicated.

LEGEND: COPD: chronic obstructive pulmonary disease, CRQ: chronic respiratory disease questionnaire, FEV_1_: forced expiratory volume in 1 second, FVC: forced vital capacity, HADS: Hospital Anxiety and Depression Scale, ILD: interstitial lung disease, mMRC: modified Medical Research Council, 6MWD: distance walked on 6-min walk test, TLCO: transfer factor for carbon monoxide.

**Table 2.** Digital devices familiarity.

	*n* (%)
Owns a smart phone, tablet or computer	38 (100)
Access to the internet	37 (97)
Frequency of Internet access	
At least once a day	35 (92)
Every few days	0 (0)
Once a week	0 (0)
A few times a month	1 (3)
Once or month or less	2 (5)
Typical location when accessing internet	
Home	37 (97)
Family/friends house	0 (0)
Library	0 (0)
Other	0 (0)
Not stated	1 (3)
Has an email address that checks regularly	37 (97)
Typical reasons for using the Internet	
Research related to health information	32 (84)
Communicate with healthcare professionals	27 (71)
Communicate with others about health-related issues	13 (34)
Research other things/topics of interest	35 (92)
Send/receive email	36 (95)
Online shopping	30 (79)
Banking/paying bills	31 (82)
Reading the news/papers/magazines/books	30 (79)
Play games online	19 (50)
Watch videos (including YouTube)	26 (68)
Use social networking websites and/or dating sites (e.g. Facebook.com)	19 (50)
Difficulty finding a Web site or searching for information	
Always easy	27 (71)
Sometimes easy	7 (18)
Not so easy	2 (5)
Difficult	0 (0)
Very difficult	0 (0)
Not stated	2 (5)
Physical restrictions that make accessing the internet difficult	
Difficulty sitting for long periods of time	2 (5)
Eyes tire easily	1 (3)

**Figure 1.**
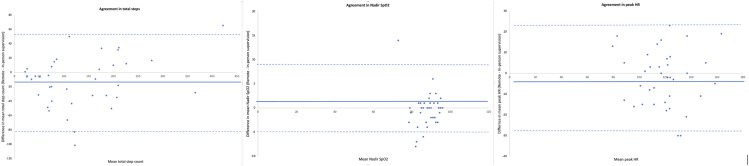
Bland-Altman plots for agreement between A. Total steps; B. Nadir SpO2; C. Peak HR during MIST supervised remotely and in-person. Solid line represents the mean difference between testing occasions. The dashed lines represent the limits of agreement.

18 participants undertook remotely supervised MIST first. Mean duration between MIST testing occasions was 7 (4) days. There was excellent agreement between tests for total step count (ICC_2,1_ 0.925, 95%CI 0.820 to 0.956). Greater step count statistically favoured in-person testing, with wide limits of agreement [Figure 1; Figure S3], however the mean difference between groups did not exceed the minimal important difference (mean difference (MD): 12 steps, 95% CI 1 to 24) (Table 3). There was very good agreement, and no difference between tests, for nadir oxygen saturation (ICC_2,1_ 0.797, 95%CI 0.643 to 0.889) and peak heart rate (ICC_2,1_ 0.782, 95%CI 0.620 to 0.880) (Table 3). Perceived breathlessness and exertion were not different between tests (Table 3). Although higher for remote MIST supervision, the number of tests where the clinician determined test cessation was not significantly different between tests (*n* = 18 vs *n* = 11, *p* = 0.4). Clinician determined test cessation was most commonly due to participant inability to keep pace with the audio recording (remote: *n* = 12; in-person *n* = 7) and oxygen desaturation below 80% (remote: *n* = 6; in-person *n* = 4). No adverse events were recorded. The total clinician encounter time, from commencement to conclusion of testing condition, was longer for remote supervision (MD 6 minutes, 95% CI two to 9). This can largely be attributed to nearly half of participants (*n* = 18, 47%) requiring telephone call support to set up their equipment for remote testing, with the majority of these calls (*n* = 10, 56%) to assist with audio speaker set up.

**Table 3.** Step test outcomes.

	In-person	Remote	In-person vs. Remote
Number of steps Mean (SD)	138 (90)	126 (99)	MD 12 95%CI 1 to 24^ϕ^
Test duration, seconds Mean (SD)	455 (211)	418 (230)	MD −37 95% CI −65 to −8^ϕ^
Nadir SpO_2_, % Median [IQR]	89 [86 to 93]	89 [84 to 93]	Z = −1.2, *p* = 0.24^γ^
Peak HR, beats/minute	120 (21)	118 (20)	MD −2 95%CI −6.3 to 2.4^ϕ^
Breathlessness at end test, borg score Median [IQR]	4 [3 to 4]	4 [3 to 5]	Z = −1.7, *p* = 0.09^γ^
Change in breathlessness (baseline to end test), borg score Mean (SD)	3 (1)	3 (2)	MD 0.1 95%CI −0.4 to 0.5^ϕ^
Fatigue at end test, borg score Mean (SD)	13 (2)	13 (2)	MD −0.04 95%CI −0.6 to 0.5^ϕ^
Change in fatigue (baseline to end test), borg score Mean (SD)	5 (2)	5 (2)	MD −0.3 95%CI −1.0 to 0.3^ϕ^
Test ceased by clinician *n* (%)	11 (29%)	18 (47%)	χ = 0.7, *p* = 0.408
Total encounter duration, minutes Mean (SD)	21 (5)	27 (9)	MD 6 95% CI 2 to 9^ϕ^

LEGEND: MD mean difference; *n* number; χ Chi square test; SpO_2_ peripheral oxygen saturation; SD standard deviation.

^γ^ = Wilcoxon signed rank test.

^ϕ^ = Paired samples *t* test.

Participation satisfaction with use of telehealth was high across all domains (Table 4). Voice/audio quality was different between tests and rated better with in-person supervision (in-person: mean 3.8 (0.4), range 3-4 versus remote: mean 3.6 (0.6), range 1-4; *p* = 0.03); leading to higher ratings of participant comfort with equipment for in-person testing (Table 5). Participant rating of confidence was not different between testing conditions (Table 5). While in-person supervision of test performance was preferred (*n* = 16, 42%), nearly as many participants had no preference for test location (*n* = 15, 40%); and one in five participants preferred remote testing (*n* = 7, 18%). No participants considered remote supervision of exercise testing to be unacceptable in the event in-person supervision was unavailable (‘If in-person supervision was not possible, would remote testing be acceptable?’: yes *n* = 33, 87%; maybe *n* = 5, 13%).

**Table 4.** Telehealth satisfaction scale (TeSS).

Participant satisfaction	In-person	Remote	In-person vs. Remote^γ^
The voice quality during the equipment	3.8 (0.4) [3-4]	3.6 (0.6) [1-4]	Z = −2.2, *p* = 0.03
The visual quality during the equipment	3.9 (0.2) [3-4]	3.9 (0.3) [3-4]	Z = −1.0, *p* = 0.3
Your personal comfort during the test	3.8 (0.4) [3-4]	3.7 (0.5) [2-4]	Z = −0.91, *p* = 0.37
Ease of getting to the testing location/logging in	3.8 (0.5) [2-4]	3.6 (0.6) [2-4]	Z = −1.66, *p* = 0.10
The length of time of the test	3.8 (0.4) [3-4]	3.5 (0.6) [2-4]	Z = −1.79, *p* = 0.07
The explanation of the test by the pulmonary rehabilitation staff	3.9 (0.2) [3-4]	3.9 (0.3) [3-4]	Z = −1.0, *p* = 0.32
The thoroughness, carefulness and skilfulness of the pulmonary rehabilitation staff	4.0 (0) [4-4]	4.0 (0.2) [3-4]	Z = −1.0, *p* = 0.32
The courtesy, respect, sensitivity and friendliness of the pulmonary rehabilitation staff	4.0 (0) [4-4]	4.0 (0) [4-4]	Z = 0.0, *p* = 1.0
How well your privacy was respected	3.9 (0.2) [3-4]	3.9 (0.2) [3-4]	Z = 0.0, *p* = 1.0
How well the staff answered your questions about the equipment (and/or test)	4.0 (0) [4-4]	3.9 (0.5) [1-4]	Z = −1.0, *p* = 0.32

Data are mean (SD) [range]. Likert scale ratings were: 1 = Poor; 2 = Fair; 3 = Good; and 4 = Excellent. ^γ^ = Wilcoxon signed rank test.

**Table 5.** Participant confidence and comfort for test conditions.

	In-person	Remote	In-person vs. Remote^γ^
How confident did you feel performing the test?	4.6 (0.6) [3 to 5]	4.7 (0.5) [3 to 5]	Z = −0.973, *p* = 0.331
How comfortable did you feel using the equipment?	4.8 (0.4) [4 to 5]	4.6 (0.7) [3 to 5]	Z = −2.310, *p* = 0.021

Data are mean (SD) [range]. Likert scale ratings from 1 (not confident at all) to 5 (very confident). ^γ^ = Wilcoxon signed rank test.

## Discussion

In this single centre cohort study, MIST supervised remotely via video-conferencing was safe, reliable and acceptable to people with chronic respiratory disease. A difference in performance of 12 steps was seen in favour of in-person supervision. While this is less than the established minimal important difference,^13^ the reference range (95% confidence interval) indicates that for some participants there could be a clinically meaningful difference in performance between testing conditions. This is important as initial exercise prescription in pulmonary rehabilitation is ideally based upon exercise test evaluation. However, as when training intensity is prescribed from more established tests of functional exercise capacity for example 6-minute walk test, monitoring of symptoms and performance during all exercise training sessions is necessary to ensure delivery of an adequate training stimulus.^2^ Beyond total steps, there were no differences in monitored physiological parameters between in-person and remotely supervised tests. Around half of participants required additional telephone support to set up equipment for the remotely supervised test; despite this, participants considered remote testing acceptable and were equally comfortable and confident with in-person and remotely supervised testing conditions.

A centre-based assessment is presently considered an essential component for the assessment and delivery of pulmonary rehabilitation,^8^ however this may serve as a barrier to some patients being able to attend an assessment and, by extension, undertake pulmonary rehabilitation. Remote administration of patient reported outcomes, including the COPD Assessment Test and St George’s Respiratory Questionnaire, have been demonstrated to be a valid and reliable alternative approach to in-person test administration for monitoring symptoms and health-related quality of life.^24^ However, remote assessment of exercise capacity has been limited to tests from which exercise training is unable to be prescribed,^9,25^ or tests that could not be administered according to international standards (i.e. course length) in the remote testing location.^11^ In demonstrating the safety and reliability of the MIST – a valid, reliable and responsive test of exercise capacity from which exercise training can be prescribed^13^ – under remote supervision, the potential to effectively deliver all elements of a pulmonary rehabilitation intervention remotely may now be realised. This has important implications for both patients and the health system, with individuals undertaking remotely delivered pulmonary rehabilitation 5 times more likely to achieve program completion (Odds Ratio 5.36, 95%CI 3.12 to 9.21) than those attending a centre-based program;^7^ which in turn is associated significantly reduced risk of hospitalisation (Hazard Ratio 0.439, *p* = 0.02) and thus healthcare expenditure.^6^

While the ability to assess exercise capacity for pulmonary rehabilitation remotely may support broader access to rehabilitation services, it is not a suitable assessment modality for all patients. There is a well-recognised digital divide particularly affecting older adults where a lack of skills, knowledge and experience coupled with limited access to devices, software and the Internet contribute to inequality and vulnerability in accessing health services remotely.^26^ In this study, familiarity with devices and access to the Internet was high, however more than half (71%) of all patients screened for this trial were ruled ineligible primarily due to language and mobility issues. People from culturally and linguistically diverse backgrounds who require an interpreter for healthcare interactions are 66% less likely to make use of services delivered via telehealth than their English-speaking counterparts.^27^ Likewise, reduced physical function and frailty can serve as a significant barrier to pulmonary rehabilitation attendance.^28^ This highlights the challenges of identifying an appropriate test of exercise capacity, compatible with remote delivery, that would be suitable for the majority of people referred to pulmonary rehabilitation. At present, clear guidance for determining patient suitability and capacity to receive pulmonary rehabilitation via telehealth remains elusive.

A key concern of remotely supervised exercise testing is participant safety.^29^ Although the sample size evaluated in this study is only modest (*n* = 38), it is larger than all previous evaluations of remote exercise test assessments undertaken in people with chronic respiratory disease.^10,11^ While no adverse events were recorded in this or previous studies, the small cumulative sample size across all studies in the area to date reinforces the need for appropriate screening and safety procedures to be in place to minimise risk of adverse outcomes for patients and clinicians. Reassuringly, when safety protocols are established a priori, the incidence of adverse events in remotely delivered exercise interventions is low. In a real-world evaluation of remotely delivered exercise training in older adults with chronic conditions, including individuals requiring oxygen, of 439 remote exercise sessions adverse events were reported on six occasions (incidence rate 1.4% of all sessions).^30^ Continuous monitoring of physiological parameters during remotely supervised exercise testing and training can further support participant safety. In the present study, there was very good agreement between tests for nadir oxygen saturation, although more tests were ceased by the health professional under remote testing conditions than with in-person supervision. While this was primarily attributable to the determination that participants were unable to keep pace with the audio recording, which may reflect audio quality or audio lag via video-conferencing, it could also reflect the lower level of confidence health professionals express when delivering care remotely as compared to in-person.^31^ Supporting patients to access the model of pulmonary rehabilitation delivery best suited to them through shared decision making and clinical implementation of a variety of evidence-based pulmonary rehabilitation program models may help to improve rehabilitation uptake and completion, and counter perceptions that telehealth delivered care is inferior to in-person consultation.^32^ In this study, to support safety, participants were required to have a suitable adult at home during remote test performance. While this requirement is not uncommon,^11^ given that people with chronic respiratory disease are more likely to be single and live alone,^33^ if the presence of a support person is considered necessary in a clinical context, this may serve to limit the utility of remote exercise testing.

Evaluation of an incremental test of functional exercise capacity is a strength of this study. As compared to self-paced tests, incremental tests are useful for eliciting a near or maximal response across a range of disease severities and are well suited to gauging response to exercise interventions using an equivalent exercise intensity pre- and post-intervention.^34^ Whether our results from the MIST would be consistent for remote supervision of other incremental exercise tests remains to be established, and feasibility may be influenced by test specific factors such as equipment requirements (e.g. cardiopulmonary exercise testing) and ability to visualise remotely (e.g. incremental shuttle walk test). Additional strengths of this study are the remote evaluation of a test of exercise capacity from which exercise training can be prescribed. The MIST, and our remote supervision protocol, makes use of commercially available equipment, occupies a small footprint, and demonstrated that participants were able to undertake the entirety of equipment set up and testing without the in-person presence of a health professional.^29^ We did, however, supply all equipment for remote testing from existing resources, with the exception of mobile data for Internet access (total data cost AUD$369 over 18-month). While this ensured standardisation of testing procedures and equipment operation, costs associated with equipment purchase and delivery and collection of equipment from patient’s homes may affect feasibility in some contexts. For remote assessment of exercise capacity to become an accepted feature of clinical practice, protocol adaptations relative to local context may be necessary to support implementation such as requiring patients to make use of their own WiFi.^35^ Whilst there are inherent costs associated with the delivery of remote healthcare, this may be offset by patient benefits, wider access to care and downstream prevention of hospitalisation and healthcare utilisation.^36^

Of note, participants rated audio interaction during remote testing more poorly than with in-person test supervision, and equipment setup relating to audio-speaker configuration posed the most significant technical challenge. An update to the MIST audio recording after trial conclusion has ameliorated the need for audio speakers in our testing set-up which may serve to reduce equipment issues associated with remote testing.

The MIST can be safely and reliably administered with remote supervision in people with chronic respiratory disease. Remote supervision of exercise capacity was acceptable to patients, who felt equally comfortable and confident in the supervisory arrangement and management of equipment with remote and in-person testing conditions. The ability to assess exercise capacity remotely, with a test that allows for accurate prescription of exercise training, may help to broaden the suite of rehabilitation options available to patients thereby supporting more equitable service access.

## Supplemental Material

Supplemental Material - Remote assessment of exercise capacity in adults with chronic respiratory disease: Safety, reliability and acceptability Supplemental Material for Remote assessment of exercise capacity in adults with chronic respiratory disease: Safety, reliability and acceptability by Narelle S Cox, Simone Dal Corso, Angela T Burge, Janet Bondarenko, Jaycie Perryman and Anne E Holland in Chronic Respiratory Disease
