# Supplementary material for: Remote assessment of exercise capacity in adults with chronic respiratory disease: Safety, reliability and acceptability
Source: Chron Respir Dis. 2025 Jan 29;22:14799731251318033. doi: 10.1177/14799731251318033 (PMC11780657; doi:10.1177/14799731251318033)
Supplement: Supplemental Material - Remote assessment of exercise capacity in adults with chronic respiratory disease: Safety, reliability and acceptability [file sj-pdf-1-crd-10.1177_14799731251318033.pdf]

Remote assessment of exercise capacity in adults with chronic respiratory disease: Safety, reliability and acceptability

NS Cox, S Dal Corso, Angela T Burge, J Bondarenko, J Perryman, AE Holland

Supplementary material

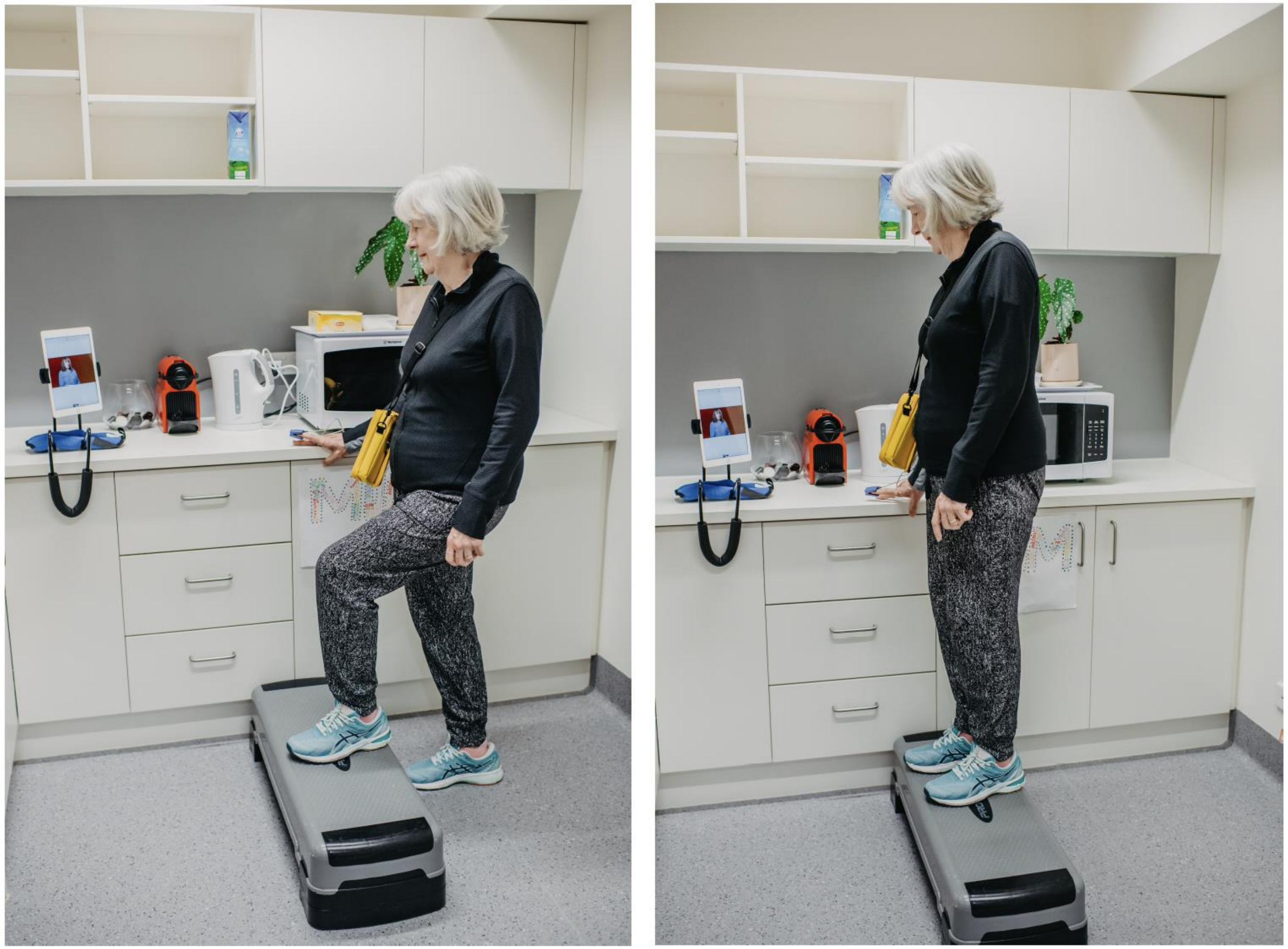

Figure S1. Example of equipment set up for remote supervision of MIST. Participants encouraged to set up equipment in a clear space, devoid of rugs and trip hazards, alongside their kitchen bench to allow for a stable surface for hand support during test performance.

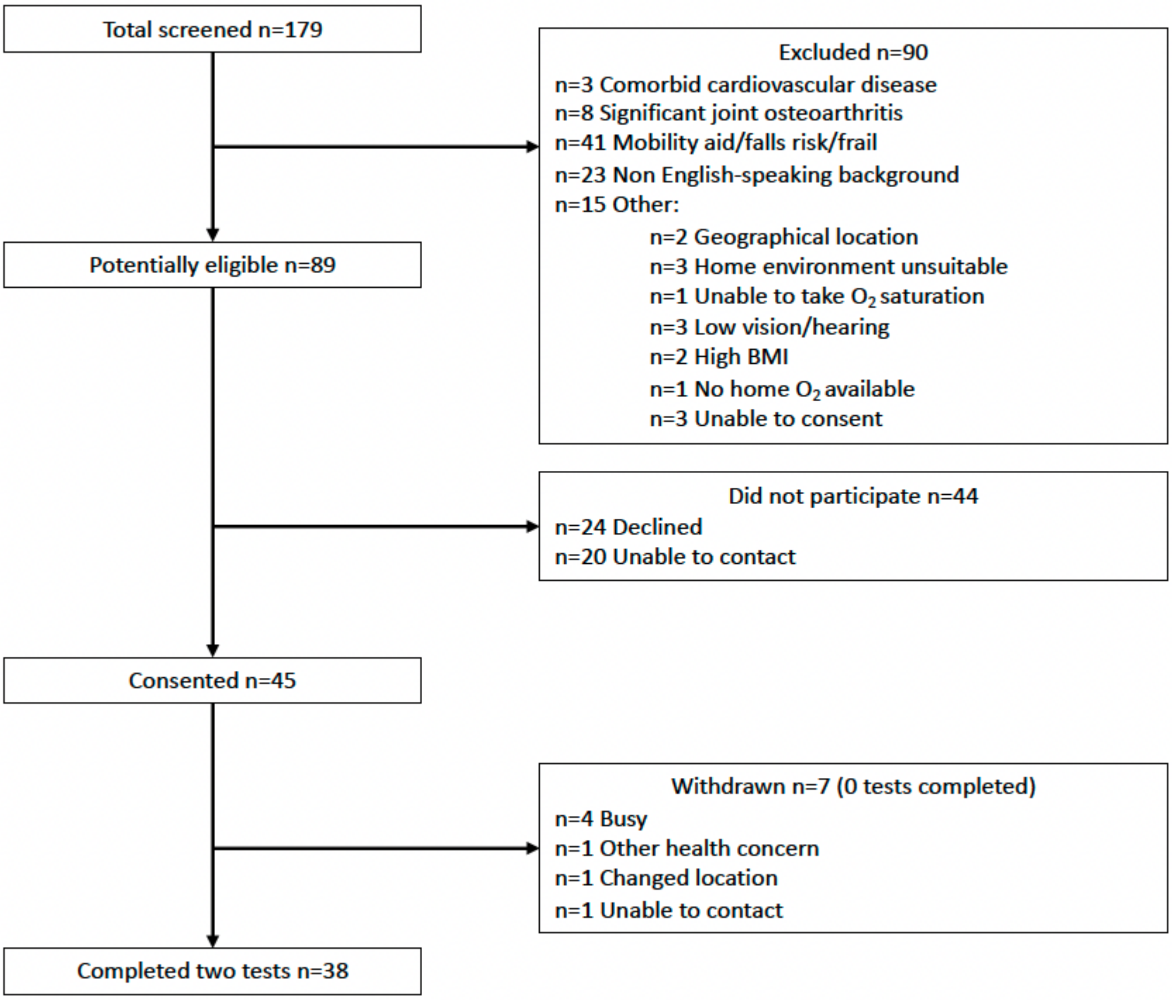

Figure S2. Recruitment flow

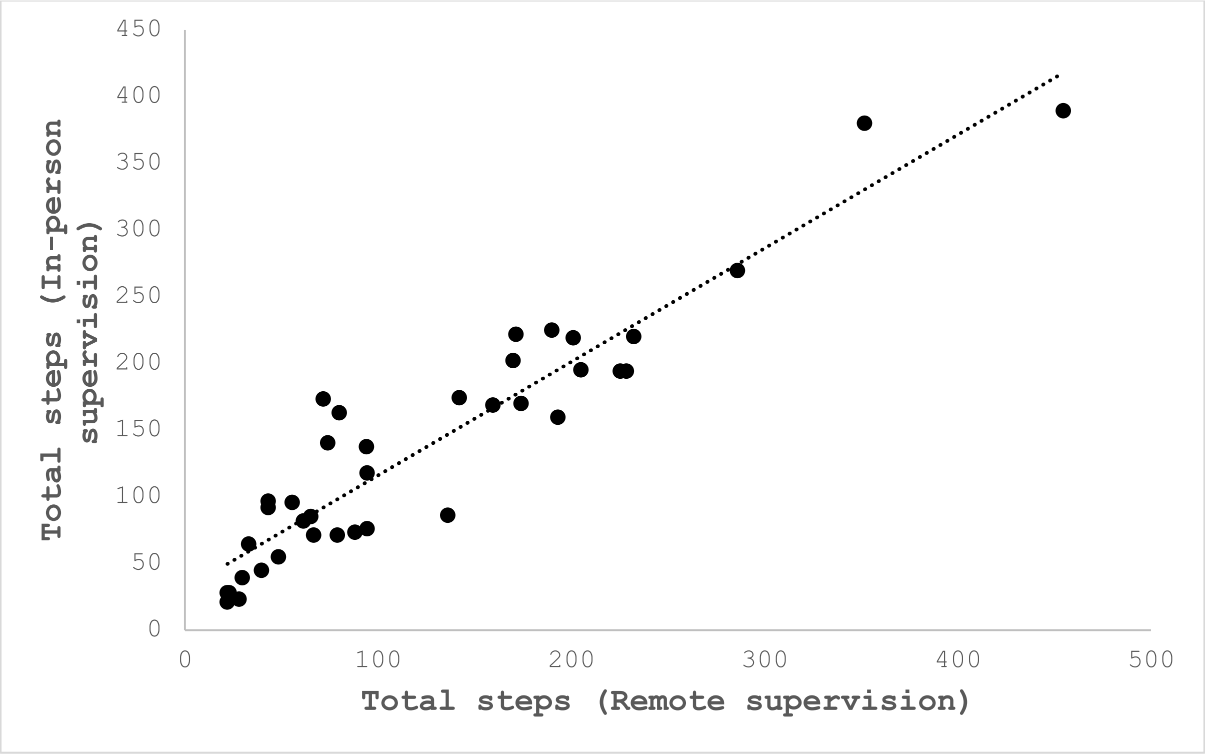

Figure S3. Total step count by testing condition
